# Spermidine/spermine *N1*-acetyltransferase-1 as a diagnostic biomarker in human cancer

**DOI:** 10.4155/fsoa-2018-0077

**Published:** 2018-09-10

**Authors:** Andrew W Maksymiuk, Daniel S Sitar, Rashid Ahmed, Brian Cheng, Horacio Bach, Rushita Adhikari Bagchi, Nina Aroutiounova, Paramjit S Tappia, Bram Ramjiawan

**Affiliations:** 1 Cancer Care Manitoba, Winnipeg, Manitoba, R3E 0V9, Canada; 2 Department of Internal Medicine, Rady Faculty of Health Sciences, University of Manitoba, Winnipeg, Manitoba, R3A 1R9, Canada; 3 Department of Pharmacology & Therapeutics, Rady Faculty of Health Sciences, University of Manitoba, Winnipeg, Manitoba, R3E 0T6, Canada; 4 BioMark Diagnostics Inc., Richmond, British Columbia, V6X 2W8, Canada; 5 Division of Infectious Diseases, Faculty of Medicine, University of British Columbia, Vancouver, British Columbia, V5Z 3J5, Canada; 6 Department of Physiology & Pathophysiology, Rady Faculty of Health Sciences, University of Manitoba, Winnipeg, Manitoba, R3E 0J9, Canada; 7 Asper Clinical Research Institute & Office of Clinical Research, St. Boniface Hospital, Winnipeg, Manitoba, R2H 2A6, Canada

**Keywords:** acetylation, amantadine, biomarkers, cancer diagnostics, cancer screening, spermidine/spermine *N1*-acetyltransferase-1

## Abstract

**Aim:**

SSAT-1 is an enzyme that plays a critical role in cell growth. Amantadine, a FDA-approved antiviral drug, is a substrate for SSAT-1. The utility of amantadine as an agent to demonstrate elevated SSAT-1 activity linked to cancer was conducted.

**Results:**

High levels of SSAT-1 expression were measured in tumor human cell lines, and in breast, prostate and lung tumor tissue. An increase in the urinary levels of acetylated amantadine in cancer patients was observed.

**Conclusion:**

Increases in SSAT-1 contents in tumor tissue could be of value in targeting cancers with high SSAT-1 expression for confirmation/quantification. The high levels of acetylated amantadine could be used as a simple and useful screening test for the presence of cancer.

## Lay abstract

In response to cancer, cells tend to overproduce specific enzymes as a self-defense mechanism. By using a safe and reliable method to capture and measure the excess enzyme spermidine/spermine N1-acetyltransferase-1, the presence of cancer can be established. This study describes a novel approach of detecting and screening cancer noninvasively in the urine of cancer patients using a safe and approved drug called amantadine that acts as a smart-tracking agent. Higher levels of the acetylated form of amantadine are detectable in the urine of cancer patients, which may serve as a detection tool. In addition, increases in the amount of spermidine/spermine N1-acetyltransferase-1 in tumor tissue may provide a tool for determining the presence of cancer during pathology assessment.

## Graphical abstract

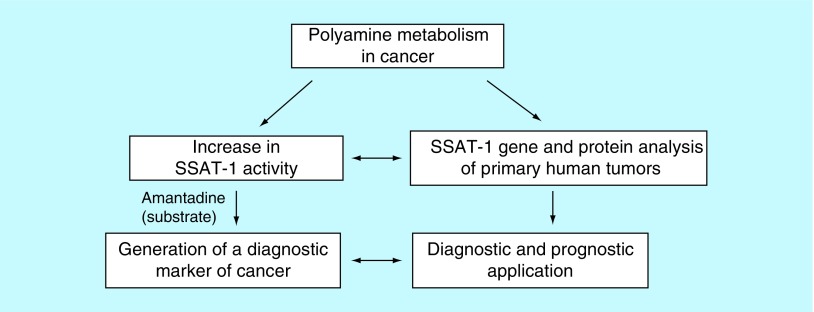

According to the American Cancer Society [1], one in seven deaths worldwide is due to cancer. Cancer is the second leading cause of death in high-income countries (following cardiovascular diseases) and the third leading cause of death in low- and middle-income countries (following cardiovascular diseases, and infectious and parasitic diseases). Excluding non-melanoma skin cancer cases, the International Agency for Research on Cancer has estimated that there were 14.1 million new cases of cancer in the world in 2012, of which 8 million occurred in developed nations. The corresponding estimates for total cancer deaths in 2012 were 8.2 million (about 22,000/day). By 2030, the global burden of cancer is expected to be 21.7 million new cases and 13 million cancer deaths.

Lung cancer is by far the leading cause of cancer death among both men and women, accounting for a 25% of cancer deaths. Each year, more people die of lung cancer than of colon, breast and prostate cancers combined. According to the Canadian Cancer Society [2], cancer is the leading cause of death in Canada and is responsible for 30% of all deaths, with an estimated 202,400 new cases and 78,800 deaths for the current year. In 2015, it was estimated that >20,000 Canadians died from lung cancer. Prognosis and survival depend on many factors; however, early and accurate diagnosis/prognosis plays a critical role in the treatment outcome and quality of life.

Several factors discourage the implementation of cancer screening for detection of early-stage cancer for the population in general. Two principal barriers are access to screening and its cost. These barriers could be overcome with a simple, accurate, reproducible and inexpensive test on a yearly basis as a general screening tool.

SSAT-1 is involved in the homeostasis of the polycationic aliphatic amines spermine and spermidine. These polyamines have multiple functions in eukaryotic cells, such as maintaining the membrane potential, and controlling intracellular pH and cell volume. Both polyamines regulate inflammatory processes, lipid metabolism, cell growth, proliferation and death [3,4]. The upregulation of SSAT-1 in different types of cancer is well documented [5–7]. Our laboratory discovered that the antiviral agent, amantadine, is a specific substrate for acetylation by SSAT-1 [8,9]. Thus, amantadine can be used to determine SSAT-1 cellular activity by measuring excretion of *N*-acetylamantadine (AA), which may indicate the presence of cancer.

SSAT-1 appears to be ubiquitous in mammalian tissues. While it is present in very small amounts in normal healthy cells, SSAT-1 can be induced by a number of factors, including toxic agents, hormones, drugs and growth factors [4,10–12]. Indeed, in human prostate cancer, there is increased expression of SSAT-1 to prevent polyamine concentrations from reaching levels that would be toxic to the cell [13]. The increased production of polyamines in cancer results in increased levels of polyamines and N1-acetylspermidine, reflecting increased SSAT-1 activity [14,15]. Indeed, an increase in monoacetylated polyamines has been detected in human and animal tumor cells/tissue [16–19].

In this study, the SSAT-1 expression levels in human normal and tumor cell lines as well as in primary patient-derived tumor tissues were assessed. Results demonstrated that high SSAT-1 expressions are present in specific cancers and the elevated SSAT-1 activity measured as the excretion of AA in urine could serve as a diagnostic test for cancer in humans.

## Materials & methods

### Cell culture

Normal human bronchial epithelial cells (ATCC^®^ PCS-300-010™, VA, USA), prostate epithelial cells (ATCC PCS-440-010™), mammary epithelial cells (ATCC PCS-600-010™), A549 (human lung tumor cells, ATCC CCL-185™), LNCaP (human prostate adenocarcinoma cells, ATCC CRL-1740™) and T-47D (human breast tumor cells, ATCC HTB133™) were used. Cells were cultured in appropriate cell growth kit, supplemented with additional growth factors as provided by ATCC, and maintained in a 5% CO_2_ humidified incubator at 37°C.

### Transcript analysis by qRT-PCR

Research and ethics approval was obtained from the University of Manitoba Research Ethics Board (Ethics File # HS 15822 [H2012:334]) prior to the study. Total RNA was extracted from tumor tissue (obtained from the Manitoba Tumor Bank, CancerCare Manitoba, Winnipeg, Canada) or from human cancer cell lines using Qiagen QIA Shredder Kit and RNeasy Mini Kit (Qiagen, ON, Canada). The RNA concentration in each sample was confirmed by nanodrop measurement. The RNA integrity was evaluated by measurement of the RNA integrity number (absorbance ratio at 260/280 nm of around 2.0). The *SSAT-1* expression was determined by qRT-PCR using a cDNA probe specific for the gene (forward: 5′-TCATCACGAAGAAGTCCTCAAG-3′ and reverse: 5′- AGCACCCCTTTTACCACTG-3′, Integrated DNA Technologies, IA, USA) using Qiagen QuaniTect SYBR Green RT-PCR kit (Qiagen). The mRNA expression levels of the human housekeeping gene *GAPDH* (and *hPRT1* in the experiments with human cancer cell lines) was measured in parallel using the corresponding PCR primers for this gene. The *SSAT-1* expression levels were normalized with *GAPDH* and/or *hPRT1* as the internal reference. Normalized *SSAT-1* expression was further analyzed by the ΔΔct method.

### SSAT-1 immunoblotting

Crude homogenate proteins (20–40 μg protein/sample) from four different primary tissues for each lung, prostate and breast cancers were resolved on SDS-PAGE (15%, polyacrylamide) and transferred onto a polyvinylidene fluoride membrane (Pall Canada, ON, Canada). After blocking with 3% skimmed milk in phosphate-buffered saline (PBS), the membrane was probed with 1:2000 primary monoclonal antibody against SSAT-1 (OriGene Technologies, MD, USA). After washing with PBS-Tween 0.05% ×3, polyvinylidene fluoride membranes were incubated with horseradish peroxidase-conjugated goat antimouse secondary antibody for 1 h at room temperature, following washing with PBS-Tween ×3. Protein bands were visualized by enhanced chemiluminescence (NEN Life Sciences, MA, USA). Mouse antitubulin (1:4000; Cedarlane, ON, Canada) was used as a control and the data were expressed as a ratio to the housekeeping protein.

### Regulatory & institutional review board approvals

Ethics approval was obtained from the University of Manitoba Research Ethics Board (ethics file #: B2003:089) prior to study implementation. The *in vivo* human study protocol was reviewed and approved by Health Canada (file # 9427-U0304-22C); notice of authorization dated 3 June 2003) and was also listed on the NIH Clinicaltrials.gov website (identifier: NCT00755898). Clinical studies were completed under GCP and GLP conditions in accordance with the standards established by the Canadian Tri-Council Policies, following approval by the University of Manitoba Research Ethics Board and Health Canada.

### Experimental subjects

In the first study, 99 patients from the CancerCare Manitoba outpatient clinics at various stages of treatment and 51 healthy adult controls provided a signed informed consent for participation. Volunteers aged >18 years were included in the study. Exclusion criteria were declared as follows: alcohol consumption within 5 days of amantadine ingestion, previous adverse reaction to amantadine and currently pregnant or lactating. On the day of the study, participants were requested to orally ingest 200 mg amantadine capsules (mylan–amantadine, amantadine hydrochloride, United States Pharmacopeia) 2 h after supper and to collect urine for 12-h post amantadine ingestion for AA analysis.

### Analytical procedures

Urine was analyzed for AA by established and validated GLP-compliant HPLC methods using d3-acetylamantadine as the internal standard for quantitation at Biopharmaceuticals Research Inc. (Vancouver, BC, Canada). Health Canada authorized Biomark AA assay standard under application number: 229838 on 7 October  2014 (investigational testing authorization).

During the development of the LC/MS/MS assay for the quantitation of AA and amantadine in human urine, calibration standards were prepared over the concentration range from 0.1 to 100 ng/ml for AA and 0.08 to 24 μg/ml for amantadine plus blank controls, based on a volume of 1 ml human urine. Quality control samples in human urine were prepared at 0.4, 4, 20, and 80 ng/ml for AA and 0.32, 3.2, and 16 μg/ml concentration levels for amantadine. All calibration standards, quality control samples and test samples were spiked with the internal standard (IS), *N*-acetyl-d3-amantadine, and processed by liquid–liquid extraction. Samples were analyzed using HPLC on a Synergi Hydro-RP 80Å 4 μm (50 × 2.0 mm, id; Phenomenex, CA, USA) column, with tandem MS/MS detection using an electrospray ionization triple-quadruple mass analyzer (Agilent 1100, Agilent Technologies, CA, USA). Positively charged matrix factors and IS ions were monitored using the multiple reaction monitoring mode. Quantitation of *ex vivo* spiked AA and amantadine in human urine was performed based on the peak area response ratio of AA or amantadine to the IS added to all samples. This LC/MS/MS assay method was successfully implemented for the measurement of AA in human urine collected from the present clinical study.

Urine creatinine was used as an estimate of completeness of the sample, in other words, normal creatinine levels indicated that the test sample is complete and appropriate for AA testing. Urine creatinine was measured by the accredited Health Sciences Centre Clinical Biochemistry Department (Winnipeg, MB, Canada).

### Data cross validation

Cross validation of findings was performed with HPLC of urine samples for the presence of AA [20,21]. Samples were coded by the study staff and the technician analyzing the biological samples did not have access to any patient information other than the code on the label.

### Statistical analysis

The concentration, total amount of AA and its excretion rate were compared using Minitab version 15.1.0.0. The data were categorized by sex, age and stage of cancer. The results were unblinded for statistical analyses. Microcal Origin version 7.5 (Origin Lab Corp., MA, USA) was used for some of the statistical analyses of the data. Some of the gene expression values as well as human volunteer data are expressed as mean ± standard error of the mean. The differences among all groups were evaluated by one-way ANOVA followed by Student's *t*-test for comparisons between two groups with Bonferroni correction for multiple analyses. A probability of p < 0.05 was considered significant.

## Results

### SSAT-1 gene expression & protein contents in tumor samples

**Figure 1.**
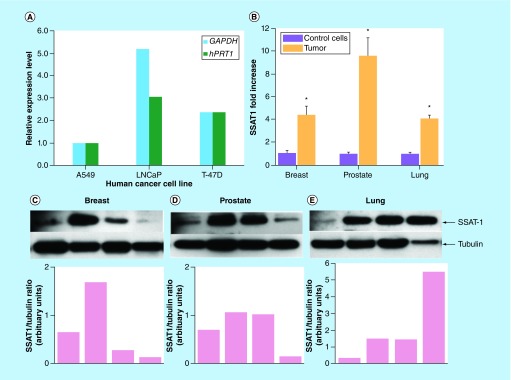
**Relative SSAT-1 gene expression in human tumor cells and human primary tumor and SSAT-1 protein contents in human tumor tissue.** The relative *SSAT-1* expression levels in **(A)** cancer cell lines using *GAPDH* and *hPRT1* as housekeeping genes and **(B)** primary human breast, prostate and lung tumor tissue normalized with *GAPDH* as measured by qRT-PCR. Data for tissue expression levels are shown as the mean ± SEM of 6–7 different primary tumor tissues. Experiments were performed in triplicate. Representative immunoblots showing 27 kDa SSAT-1 protein levels were quantified by densitometry using tubulin from four different patients for **(C)** breast tumor tissue, **(D)** prostate tumor tissue and **(E)** lung tumor tissue. Normal human bronchial epithelial cells, prostate epithelial cells and mammary epithelial cells were used as controls as described in the methods. SEM: Standard error of the mean.

*SSAT-1* gene and protein was detectable in all tumor tissue examined. Analysis of the *SSAT-1* gene expression revealed detectable levels of *SSAT-1* gene in A549 (human lung tumor cells) LNCaP (human prostate adenocarcinoma cells) and T-47D (human breast tumor cells) (Figure 1A). Furthermore, analysis of patient-derived breast, prostate and lung primary tumors revealed approximately a five- to ten-fold increase in *SSAT-1* mRNA levels, as compared with respective noncancerous normal human bronchial epithelial cells, prostate epithelial cells and mammary epithelial cells that were used as proxy controls (Figure 1B). Immunoblotting also revealed increased SSAT-1 protein contents in the corresponding tumor tissue (Figure 1C–E). It should be noted that, in view of the very low levels of SSAT-1 in normal cells, we were unable to detect SSAT-1 protein in the noncancerous human control cells. Accordingly, these western blot images have not been presented. Table 1 shows the demographics (patient age and sex, tumor laterality/site, morphology and staging) of the tumor tissue used for the gene and protein expression studies.

**Table 1.** **Demographic information of primary tumor tissue used for gene and protein expression studies.**

**Tumor tissue**	**Age (y)**	**Sex**	**Laterality/site**	**Morphology**	**Stage**
*Breast*					

1^†^	61	F	Left	Infilt. Ductal & lobular mixed carcinoma	TxN0M0

2^†^	55	F	Right	Infilt. Ductal & lobular mixed carcinoma	T2N1aM0

3	80	F	Right	Infilt. Ductal carcinoma	T2N1aM0

4	44	F	Right	Infilt. Ductal carcinoma	T2N0M0

5	43	F	Left	Infilt. Ductal & lobular mixed carcinoma	T2N2M0

6^†^	40	F	Right	Infilt. Ductal & lobular mixed carcinoma	T1cN1M0

7^†^	87	F	Right	Infilt. Ductal & lobular mixed carcinoma	T4bN3aM0

*Prostate*					

1	61	M	–	Adenocarcinoma	N/A

2^†^	65	M	–	Adenocarcinoma	T3bN1M0

3^†^	62	M	–	Adenocarcinoma	T3bN1M0

4	66	M	–	Adenocarcinoma	T3bN1M0

5^†^	50	M	–	Adenocarcinoma	T2cN0M0

6^†^	64	M	–	Adenocarcinoma	T2cN0M0

*Lung*					

1^†^	52	M	RML	Adenocarcinoma with acinar, solid pattern	T2N0M0

2^†^	75	F	RUL	Adenocarcinoma	T2N0M0

3	68	F	RUL	Adenocarcinoma	T2N0M0

4^†^	80	F	LUL	Adenocarcinoma	T2bN0M0

5^†^	69	F	LUL	Adenocarcinoma	T2aN0M0

6	55	F	LLL	Adenocarcinoma	T3N1M0

7	71	F	LUL	Adenocarcinoma	T2aN2M1b

^†^Samples used for immunoblotting.

Infilt.: Infiltrating; LLL: Left lower lobe; LUL: Left upper lobe; M: Metastasis; N: Lymph nodes; RML: Right middle lobe; RUL: Right upper lobe; T: Staging, tumor.

### Healthy control & cancer patient characteristics

**Figure 2.**
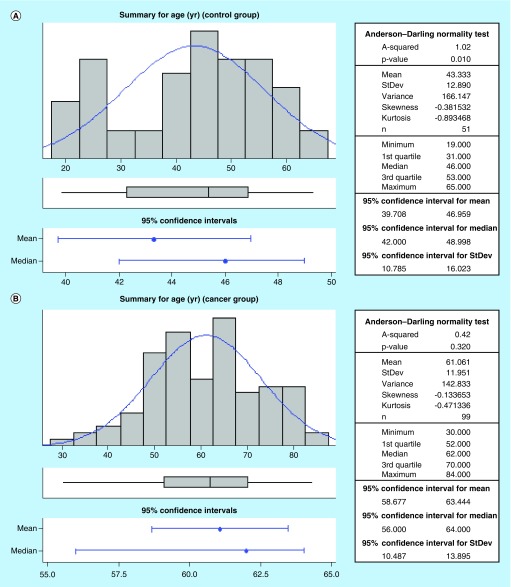
**Age distribution in healthy control and cancer patients.**

The characteristics of the participants in this study are shown in Table 2. There were no statistical differences between the age of the female (43 ± 2 years) and male (44 ± 3 years) participants in the healthy control group. Similarly, there were no statistical differences between the age of the female (62 ± 3 years) and male (61 ± 2 years) participants in the cancer group. Of note, the participants in the cancer group were older than the healthy control volunteers (Figure 2A & B). No significant differences in body weight and serum creatinine levels in the health volunteers and cancer patients were observed. Table 3 shows the different cancer types groupings as well as some staging information with a vast majority (56/99) of patients with lung cancer. Available staging information revealed 15 lung cancer patients at stage 3 and 12 patients with stage 4 disease (Table 3).

**Table 2.** **Participant demographics.**

**Parameter**	**Healthy volunteers**	**Cancer patients**
Age (y)	43 (31–53)	62 (53–70)

Males/females	23/28	58/41

Body weight (kg)	75 (64–89)	77.8 (63.5–89.8)

Urine creatinine (μmol/l)	79 (67–84)	74 (61–91)

Data presented as mean and range in parentheses.

**Table 3.** **Cancer type and staging of patients.**

**Cancer^†^**	**Patients (n)**	**Stage**		

		**II**	**III**	**IV**
Lung	56	1	15	12

GI	16	1	1	2

Breast	4	1	–	–

Prostate	1	–	–	–

Oral/naso	11	1	–	1

Other	11	–	2	–

Total	99	4	18	15

^†^Lung cancer includes all patients with nonsmall cell, adenocarcinoma, squamous, small cell and mesothelioma. GI cancer is all patients with colon, rectal, cecal, eosophagus and pancreatic cancers. For others, cancer type includes angiosarcoma met, brain met, non-Hodgkins lymphoma, myosarcoma met, follicular lymphoma, parotid gland, B-cell lymphoma, multiple myeloma and lymphoma. Staging information was not available for all samples.

### Urinary acetylamantadine levels in healthy & cancer patients

**Figure 3.**
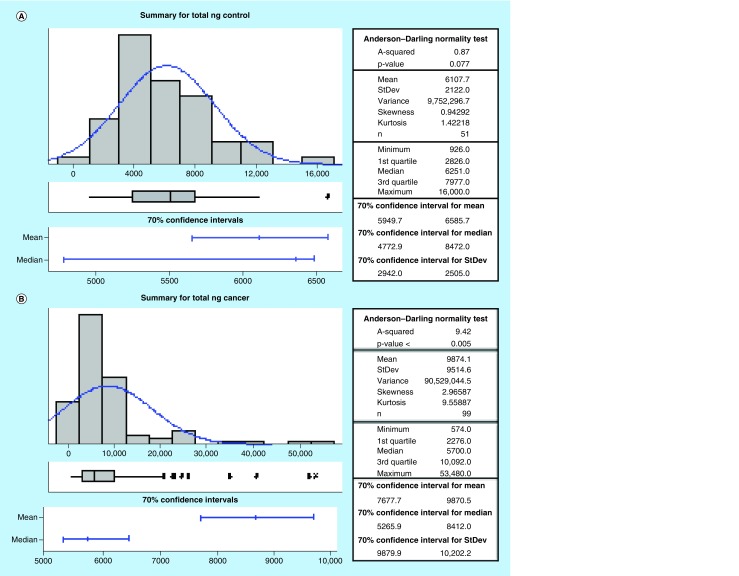

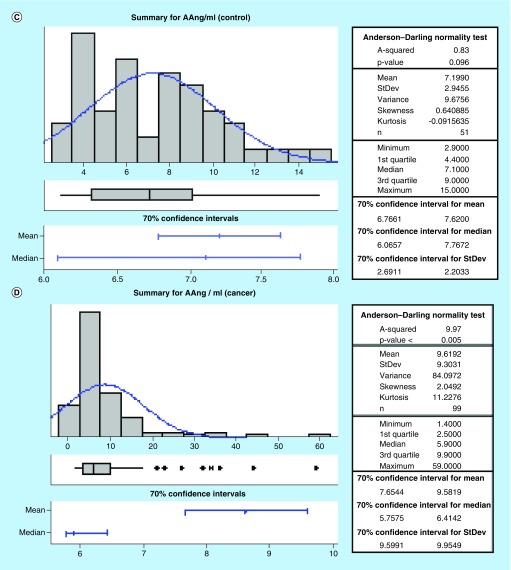

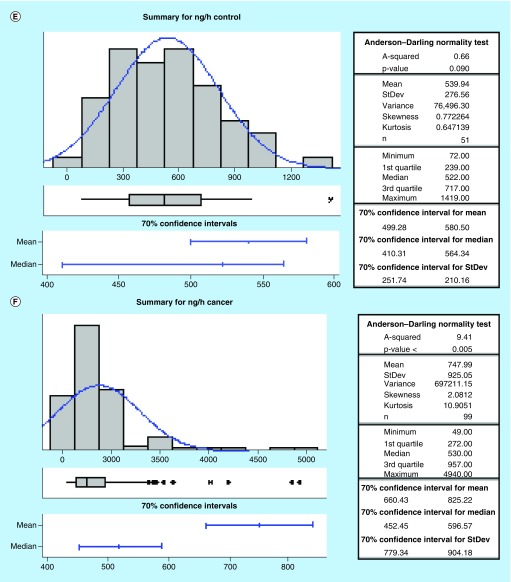
**Total concentration and excretion rates for urinary acetylamantadine as determined by LC/MS/MS technique.** A parametric method was used to analyze the data for total AA (in ng amount) in **(A)** healthy controls and **(B)** cancer patients as well as for the AA concentration in **(C)** healthy controls and **(D)** cancer patients. Values are expressed as ng/ml. The excretion rate was calculated by total ng amount of AA divided by 12 and is expressed as ng/h and is shown for **(E)** healthy controls and **(F)** cancer patients. Urine was collected over a 12-h period after the ingestion of amantadine. AA: Acetylamantadine.

**Figure 4.**
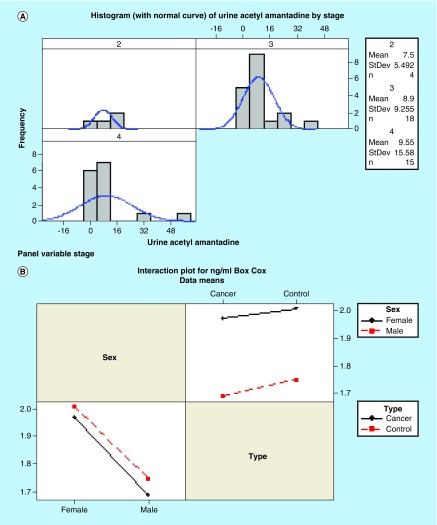

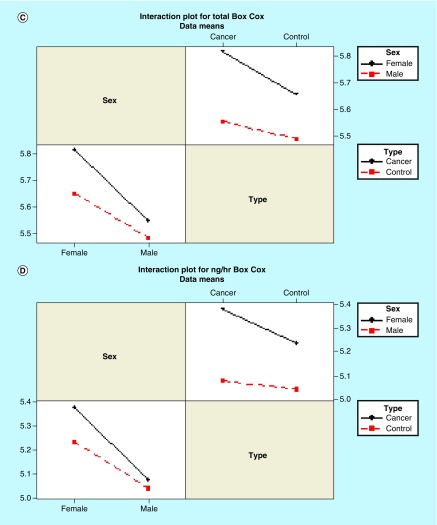
**Acetylamantadine concentration in the urine of cancer patients according to stage of disease and sensitivity of acetylamantadine detection in male and female healthy control participants and cancer patients.** Mean values for AA concentration in **(A)** cancer patients are expressed in ng/ml for all cancers stage 2 (n = 4), stage 3 (n = 18) and stage 4 (n = 15), whereas the sensitivity data is shown for **(B)** AA concentration (ng/ml), **(C)** total AA (ng) and **(D)** rate of excretion (ng/h). AA: Acetylamantadine.

All participants had detectable AA in their urine. The data revealed that the control group has a lower amount of total urinary AA as compared with the cancer group by parametric analysis (Figure 3A & B). Furthermore, cancer patients excreted AA at higher concentration and rate compared with healthy controls (Figure 3C–F). Interestingly, a gradual increase in the urinary AA concentration was measured with progressing cancer severity, in other words, stage II to stage IV (Figure 4A). Also, the SSAT-1 response was higher in females than in males. In this regard, females were observed to exhibit a greater difference in the total AA amount and rate of excretion (Figure 4B & C) as compared with male counterparts. These data suggest that females may have a higher sensitivity (cancer vs control) of detection as compared with males.

## Discussion

We, as well as others, have shown that SSAT-1 signals can be a useful marker for assessing the presence and potentially the stage/aggressiveness of cancer [8,9,14–19]. The combined approach revalidates the linear correlation between *SSAT-1* gene expression, protein level and activity.

In this study, an LC/MS/MS assay method was developed for the quantitation of AA and amantadine in human urine based on a quadratic calibration regression function. The performance of the assay for AA and amantadine in human urine was successfully qualified based on the assessment of assay specificity, selectively, linearity, accuracy, precision, quantitation range, recovery and LLOQ against predetermined assay acceptance criteria. The detection of AA by the developed method is reliable, accurate, highly sensitive and reproducible.

Results of this study suggest that using amantadine as a proxy to quantify indirectly the increased SSAT-1 acetylation activity in cancer have clinical utility. The growth of transplantable tumors is associated with an intensification of sulfadimidine acetylation. Treatment of the tumors caused a decrease in acetylation rate and when tumor suppression failed, the rate of sulfadimidine acetylation did not diminish [22]. Consistent with this finding, the level of *N*-acetylation of sulfadimidine in cancer patients is higher in both fast and slow acetylator phenotypes, compared with corresponding controls [23,24]. Comparison of phenotyped malignant and benign breast tissue showed increased acetylation of both p-aminobenzoic acid and sulfamethazine in human malignant tissue. Enzyme activity was twice as great for prototypical *N*-acetyl transferase 2 (NAT2) compared with NAT1 acetyltransferase substrates in malignant tissue [25]. Effective treatment of malignant lymphoma caused a decrease in sulfadimidine acetylation [24]. It has been assumed that the increased acetylation activity could be attributed to the acetyltransferases NAT1 and NAT2. Although it is generally accepted that NATs are constitutive enzymes and are not inducible, induction of NAT2 has been demonstrated in mouse kidney under the influence of glucocorticoid or androgen [26]. The increases in acetylation described in malignancy for NAT2-selective substrates in humans may also be explained by increased SSAT-1 activity. We have previously reported that amantadine is a specific substrate of SSAT-1 and can differentiate between acetylation by SSAT-1 and NATs, since amantadine is not a substrate for acetylation by either NAT1 or NAT2 [8,9].

Accordingly, we hypothesized that amantadine acetylation would serve as a biomarker for malignancy since it occurs only by SSAT-1, an enzyme upregulated in tumor tissue. Our hypothesis was supported by the total AA and AA/h response and suggests that amantadine acetylation has potential as a diagnostic test for cancer. The data from our Phase II proof of concept study provided the basis for designing a Phase III study that will determine the effectiveness of this biomarker in a large, untreated, newly diagnosed patient population after optimization of the urine collection time and dosage in order to understand the pharmacokinetics and pharmacodynamics of AA.

We have identified SSAT-1, an enzyme in a key metabolic polyamine pathway that is overexpressed showing high activity in cancer patients compared with healthy controls. Interestingly, SSAT-1 has a unique feature that binds very specifically to amantadine, an approved US FDA drug. Then, shortly upon ingestion of the drug, it enters the cells in the body, where it is acetylated by SSAT-1. The catalytic activity of SSAT-1 on amantadine released the product AA, which passes through the urine (noninvasive) and, is easily detected. Because the assay detects a final byproduct (AA), which is not further metabolized, the signal is higher compared with traditional cancer biomarkers. In this regard, the already known blood-based protein biomarkers such as [27–34] carcinoembryonic antigen, carbohydrate antigen 19-9 l and α-fetoprotein alone are unreliable as well as lack the sensitivity and specificity needed for early diagnosis of cancer [27–34], which underscores the importance of identifying and validating more sensitive and accurate markers [27]. It should be noted that side effects from amantadine ingestion are highly unlikely since only single doses of amantadine are administered. Any side effects, if they occur, may include insomnia, jitteriness, difficulty in concentrating and mental depression. However, these are associated with long-term ingestion of amantadine and are highly unlikely to occur with ingestion of a single dose [35,36].

It should be mentioned that the data distribution of the cancer group presented in the current study was non parametric with a very wide spread of values suggesting the presence of interfering factors. These uncontrolled confounding factors may include any ongoing treatment, other co-morbidities and the fact that all cancer patient data were pooled. On the other hand, the data distribution for the control group was parametric, suggesting a minimized contribution of any possible confounding factors. In view of the higher AA concentrations, total AA amount, AA excretion rates and the gradual increase in AA concentration with higher cancer disease severity, classification stage suggests that a strong correlation exits between advancing disease, stage of detection and SSAT-1 activity level. It is also important to note that the detection of *SSAT-1* transcripts and protein in primary human tumor tissue could also serve as a valuable prognostic and diagnostic assessment tool for testing for the presence of cancer at biopsy. Interestingly, an additional hypothesis is borne out from the present study that links increased SSAT1 levels with epigenetics, particularly DNA hypomethylation. In this regard, secondary to increased putrescine levels, as a consequence of increased SSAT1 activity, as well as with stabilization of adenomethionine decarboxylase, S-adenosylmethionine is directed for the biosynthesis of polyamines. However, with this increased consumption of S-adenosylmethionine in the polyamine pathway, there could be a reduction in the availability of methyl groups, resulting in DNA hypomethylation. DNA hypomethylation, which is a hallmark of the early phase of certain cancers [37] and nonneoplastic diseases [38], may be linked to elevated SSAT-1. This hypothesis is therefore an additional consideration for the importance of SSAT-1 in human cancer.

Although considerable advances in cancer diagnostics have been developed in the last 30 years, the overall mortality rate for most cancers has shown little improvement. Currently, the industry is dominated by imaging modalities (computed tomography, MRI and mammograms) followed by molecular and biomarker diagnostic assays. Imaging modalities are expensive, often inaccessible with the consequences of exposure to ionizing radiation. On the other hand, biopsies for molecular and biomarker diagnostics are invasive and uncomfortable. They may or may not result in the detection of the carcinoma. Limitations of these techniques have sequestered their use to post-symptomatic rather than pre-symptomatic detection.

With the recent emergence of metabolomics and the identification of specific enzymes being overregulated or overexpressed in cancer cells [39–42], it is our viewpoint that the enzyme is not the biomarker, but that the biomarker is the product (metabolite) of the enzymatic reaction. An overexpressed enzyme related to cancer will produce many times more metabolite, thereby exponentially increasing the signal-to-background noise ratio. The result is an amplified biomarker that acts like beacon for early detection that no traditional biomarker can match; however, while this remains to be proven, our findings demonstrate that the product of elevated SSAT-1 and the generation of AA as a product of its enzymatic activity are quantifiable and clinically relevant.

## Conclusion

Increases in SSAT-1 protein contents and gene expression in primary tumors is linked to a concomitant higher level of AA in urine of cancer patients upon ingestion of amantadine.

## Future perspective

While a larger study is necessary, our initial findings support further investigations to determine the utility of our amantadine test as a simple screening test for the presence of cancer as well as a surveillance test in populations at high risk for developing cancer. The elevated *SSAT-1* expression could be utilized for confirmation/quantification during pathology assessment. The multiple approaches, undertaken in the present study to quantify SSAT-1 could also serve as prognostic (staging) and diagnostic tools for different types of cancer.

**Summary points**

- SSAT-1 (gene and protein) is upregulated in human cancer.
- Amantadine is a specific substrate for acetylation by SSAT-1.
- A higher urinary level of acetylamantadine is detected in patients with different types of cancer that may also be linked to staging.
- SSAT-1 response appeared to be higher in females than in males.
- The amantadine test may represent a novel simple screening and surveillance tool for different types of cancer.
